# Asymptomatic immune responders to *Leishmania* among HIV positive patients

**DOI:** 10.1371/journal.pntd.0007461

**Published:** 2019-06-03

**Authors:** Laura Botana, Ana Victoria Ibarra-Meneses, Carmen Sánchez, Alicia Castro, Juan Victor San Martin, Laura Molina, Jose Manuel Ruiz-Giardin, Eugenia Carrillo, Javier Moreno

**Affiliations:** 1 WHO Collaborating Centre for Leishmaniasis, National Centre for Microbiology, Instituto de Salud Carlos III, Majadahonda (Madrid), Spain; 2 Hospital Universitario de Fuenlabrada, Fuenlabrada, Madrid, Spain; Pasteur Institute of Iran, ISLAMIC REPUBLIC OF IRAN

## Abstract

Concomitant infection with human immunodeficiency virus (HIV) and the *Leishmania* parasite is a growing public health problem, the result of the former spreading to areas where the latter is endemic. *Leishmania* infection is usually asymptomatic in immunocompetent individuals, but the proportion of HIV+ individuals in contact with the parasite who remain asymptomatic is not known. The aim of the present work was to examine the use of cytokine release assays in the detection of asymptomatic immune responders to *Leishmania* among HIV+ patients with no previous leishmaniasis or current symptomatology. Eighty two HIV+ patients (all from Fuenlabrada, Madrid, Spain, where a leishmaniasis outbreak occurred in 2009) were examined for *Leishmania infantum* infection using molecular and humoral response-based methods. None returned a positive molecular or serological result for the parasite. Thirteen subjects showed a positive lymphoproliferative response to soluble *Leishmania* antigen (SLA), although the mean CD4+ T lymphocyte counts of these patients was below the normal range. Stimulation of peripheral blood mononuclear cells (PBMC) or whole blood with SLA (the lymphoproliferative assay and whole blood assay respectively), led to the production of specific cytokines and chemokines. Thus, despite being immunocompromised, HIV+ patients can maintain a Th1-type cellular response to *Leishmania*. In addition, cytokine release assays would appear to be useful tools for detecting these individuals via the identification of IFN-γ in the supernatants of SLA-stimulated PBMC, and of IFN-γ, MIG and IL-2 in SLA-stimulated whole blood. These biomarkers appear to be 100% reliable for detecting asymptomatic immune responders to *Leishmania* among HIV+ patients.

## Author summary

The proportion of patients with HIV+ who have at some time been infected with the *Leishmania* parasite, but who remain asymptomatic, is unknown. It is important to be able to identify such patients to determine the prevalence of asymptomatic leishmaniasis in the HIV+ population, and because these persons are at increased risk of developing symptomatic visceral leishmaniasis. In the present work, a population of HIV+ patients showing a cellular immune response to *Leishmania infantum* was identified. These subjects all showed a clear Th1-type response when their PBMC or blood were stimulated *in vitro* with soluble *Leishmania* antigen (SLA). Cytokine release assays and the detection of IFN-γ, MIG and IL-2 (specific biomarkers for immunity to *Leishmania*) were found to be useful for detecting this population of HIV+ patients. New studies with larger numbers of patients are needed to confirm the present results.

## Introduction

Leishmaniasis is a neglected, vector-borne disease associated with high morbidity, caused by protozoan pathogens of the genus *Leishmania*. In visceral leishmaniasis (VL), the most serious form of the disease (fatal if untreated), the parasite is systemically disseminated. VL is hypoendemic in the Mediterranean region, where the causal agent is *Leishmania infantum* [1].

In 2017, nearly 160,000 people were diagnosed with HIV in the WHO European Region, marking another year of alarming new HIV numbers [2]. HIV is most prevalent in France, Spain, Italy and the United Kingdom; Spain has the highest DALYs value attributable to HIV/AIDS [3].

The number of reported cases of *Leishmania*/HIV co-infection increased rapidly during the 1990s, a consequence of the spread of the HIV pandemic, increased awareness among reporting institutions, and the growing geographical overlap between the two diseases [4].

*Leishmania/*HIV co-infection has been recognised as an emerging problem in areas endemic for leishmaniasis [5]. In co-infected patients, the symptoms of VL may be more severe than in immunocompetent individuals, relapse is more common, mortality higher, parasite loads greater, and organs not normally involved in VL may be parasitised [6]. Co-infected patients are also potential spreaders of *Leishmania*, posing a huge problem for current elimination strategies [7]. Indeed, HIV infection is recognised as an emerging challenge in the control of VL [4].

HIV infection increases the risk of developing VL between 100 and 2320 times [6, 8]. The marked fall in the number of CD4+ T cells caused by the virus, along with the reduced production of IFN-γ, and the lesser leishmanicidal capacity of the macrophages [9], results in the replication and uncontrolled dissemination of the parasite around the body. HIV infection is also associated with the reactivation of latent *Leishmania* infection and its progression towards VL [1]. Similarly, active *Leishmania* infection can increase the replication of the virus, encouraging progression to full-blown acquired immunodeficiency syndrome (AIDS) [4]. In the pre-HAART (highly active antiretroviral therapy) era, patients with HIV also infected with *Leishmania* commonly developed VL; these days, however, the efficiency of HAART allows a variable proportion of such patients to remain asymptomatic [8, 10].

The variation in the ratio of patent VL cases *versus* asymptomatic cases in different *L*. *donovani*- and *L*. *infantum*-endemic areas (from 2.4:1 in Sudan to 50:1 in Spain [11]) reflects differences in parasite virulence and host characteristics, but perhaps also in study design and the tests used to identify asymptomatic infection. Cell immunity usually remains positive for several years, sometimes even throughout an individual’s life [12, 13]. However, serological markers can revert to negative within 4 months of any first sample being inspected [14]. Serology is also unsatisfactory for detecting asymptomatic *Leishmania* infection in endemic areas where mean parasitaemia levels are low or intermittent [15]. No normalised or commercial techniques exists for defining asymptomatic *Leishmania* infection. An asymptomatic subject is usually regarded as someone from an endemic area who shows an immune response (either antibodies or a positive leishmanin skin test [LST]) against *Leishmania*, or who has parasites in the blood, but who remains healthy [15, 16].

VL is largely diagnosed using molecular and serological techniques. However, the serological detection of *Leishmania* in patients with HIV or AIDS is not very sensitive since the parasite elicits a weak antibody response [17, 18]. Molecular diagnoses are normally reliant on PCR, which is highly sensitive and specific for VL in co-infected patients [19] since the parasite load is high, even in peripheral blood. However, PCR has been used in very few studies to detect patients with HIV who are also asymptomatic but infected with *Leishmania* [20, 21].

The leishmanin skin test has been widely used in the field to study the prevalence of infection, but its side effects, and the suspect quality of its manufacture, have seen it banned in some countries, including most European nations (certainly in Spain) [17]. Cytokine release assays using whole blood or PBMC stimulated with soluble *Leishmania* antigen (SLA) are useful for monitoring patients who have undergone solid organ transplantation following treatment for VL, and for detecting asymptomatic *Leishmania* infection in this population [22]. They have also been found useful for establishing the efficacy of treatment for VL in patients also infected with HIV, and for assessing the need to maintain secondary prophylaxis in such patients [23]. Recent studies have confirmed that IFN-γ and IL-2 are good biomarkers of asymptomatic *Leishmania* infection [24], but so too are the induction protein of IFN-γ (IP-10 or CXCL10), the monokine induced by IFN-γ (MIG or CXCL9), and monocyte chemotactic protein 1 (MCP-1 or CCL2) [25, 26].

Antiretroviral treatment has been very successful in controlling HIV replication and preventing the appearance of opportunistic infections in HIV+ patients [10, 27]. HAART not only reduces viral replication but leads to an increase in the number and functionality of CD4+ T cells and reverts the majority of immunological abnormalities. In the pre-HAART era, patients co-infected with HIV and *Leishmania* commonly failed to produce immunity against the parasite following treatment for VL [28], but these days most HAART-treated patients do so [23]. This begs the question of whether, in *Leishmania*-endemic areas, there are asymptomatic immune responders among HIV+ individuals (as is seen in solid organ transplantation-associated immunodepressed patients [22]). Cell immunity techniques might be used to detect such individuals. The aims of the present work were 1) to determine whether these techniques, along with serological and molecular tests, can be used to make such identifications, and 2) to characterise the immune response against the parasite in such individuals.

## Materials and methods

### Ethics statement

This study was approved by the *Hospital de Fuenlabrada* (APR12–65 and APR14-64). All participants gave their written informed consent to be included.

### Study population

Blood was collected from 82 HIV+ adult patients at the *Hospital de Fuenlabrada* between 2015 and 2017. All lived in Fuenlabrada (Madrid, Spain), a *Leishmania infantum*-endemic area with a high prevalence of infection. All subjects were undergoing antiviral treatment and had their viral load regularly monitored. None of the subjects had shown any sign of leishmaniasis. All blood samples were subjected to several specific *Leishmania* tests (humoral, cellular and molecular). Of the subjects providing samples with negative results to all of them, 19 were randomly selected as negative controls (NC) for analysis. S1 Table provides a detailed description of the present HIV+ patients.

### Lymphocyte populations

To determine the number of circulating lymphocytes, combinations of CD4/CD8/CD3, CD3/CD19/CD45 and CD3/CD16+CD56/CD45 antibodies, conjugated with FITC/ PE/PerCP (BD Tritest, USA) respectively, were added to 50 μl aliquots of peripheral blood, and analysed by flow cytometry using FlowJo v.7.6.5 software. The percentages obtained were multiplied by the total number of lymphocytes in the haemogram to obtain absolute values for circulating lymphocytes. Values for healthy individuals were used as a reference.

### Preparation of soluble *Leishmania* antigen (SLA)

*L*. *infantum* antigen was prepared from promastigote cultures in the stationary phase (JPC strain, MCAN/ES/98/LLM-722), as previously described [29]. The parasites were first washed with 1X phosphate-buffered saline (PBS) and centrifuged at 1000 *g* for 20 min at 4°C. The supernatant was discarded and the pellet resuspended in lysis buffer (50 mM Tris/5 mM EDTA/HCl, pH 7). These samples were subjected to three cycles of freezing/thawing, and then sonicated three times (40 W for 20 s) before being centrifuged again at 27,000 *g* for 20 min at 4°C. The supernatants were collected, divided into aliquots, and stored at -80°C until use. The protein content was quantified following the Bradford method, using the Pierce BCA Protein Assay Kit (Bio-Rad, USA).

### Culturing and stimulation of peripheral blood mononuclear cells (lymphoproliferative assay [CPA])

Blood samples (10 ml) were collected in heparinised vials from all subjects. Peripheral blood mononuclear cells (PBMC) were separated out using a Ficoll-Hypaque gradient (Rafer, Spain), resuspended in complete RPMI supplemented with 10% foetal bovine serum, and cultured (in triplicate) at an initial concentration of 2x10^6^ cells/ml in 96-well plates with either complete RPMI (negative control), SLA (10 μg/ml) or phytohaemagglutinin-M (PHA-M) (5 μg/ml) [22]. All cultures were kept for 6 days at 37°C in a 5% CO_2_ atmosphere. The lymphoproliferative response of each subject was then determined by bromodeoxyuridine incorporation using the Cell Proliferation Kit (GE Healthcare Life Sciences, UK), following the manufacturer's instructions. Results were expressed in the form of a stimulation index (absorbance of stimulated cells/unstimulated cells). The culture supernatants were collected and stored at -20°C for later cytokine and chemokine analysis.

### Stimulation of whole blood with SLA (whole blood assay [WBA])

Aliquots (500 μl) of whole blood were incubated in tubes with 10 μg/ml SLA or 5 μg/ml PHA-M. A further tube with no SLA was used as a negative control. All tubes were incubated at 37°C for 24 h, as previously described [22, 24]. They were then centrifuged at 2000 *g* for 10 min. The supernatants were removed and kept at -20°C for later cytokine and chemokine analysis.

### Cytokine and chemokine determination

IFN-γ, TNF-α, granzyme B, IP-10, MIG, IL-2 and IL-10 were determined in 50 μl of supernatant from the PBMC cultures, and in the same volume of SLA-stimulated plasma from the WBA [25], using the CBA Human Soluble Protein Flex Set Capture Bead Kit (Becton Dickinson, USA), following the manufacturer's instructions. Results were captured by flow cytometry using Flow Cytometric Analysis Program Array software (Becton Dickinson, USA). Results from each cytokine and chemokine were expressed as the difference between the SLA-stimulated and control plasma concentrations.

### Enzyme-linked immunosorbent assay

An enzyme-linked immunosorbent assay (ELISA) was used to detect antibodies to SLA [22]. Briefly, 96-well plates (NuncMaxisorp Immuno Plates, USA) were coated with 100 μl/well of 10 μg/ml SLA and left overnight at 4°C. The plates were then washed three times with PBS, 0.1% Tween 20 (PBS-T), pH7.4, and blocked with 200 μl/well of PBS containing 0.1% Tween 20 and 3% BSA for 1 h at 37°C. After washing with PBS-T, diluted blood plasma (1/200 in PBS-T) was added (100 μl/well) and incubated for 2 h at 37°C. The plates were then washed with PBS-T and 100 μl/well of 1/5000-diluted HRP-conjugated anti-human Ig (Invitrogen, USA) were added for 30 min at 37°C. All plates were then developed with 100 μl/well of Sigma Fast o-phenylene diamine dihydrochloride (OPD) tablets (Sigma, USA) for 20 min. The reaction was stopped with 50 μl/well of 2NHCl, and absorbance measured at 492 nm.

### Immunofluorescent antibody titres

Immunofluorescent antibody titre (IFAT) analyses of plasma samples were performed using 2 × 10^5^ *L*. *infantum* promastigotes in PBS per well (MCAN/ES/98/LLM-722), as previously described [22]. Subject plasma was assayed as two-fold serial dilutions (from 1/20 to 1/640) in PBS to determine total IgG levels using fluorescein isothiocyanate-conjugated goat anti-human IgG (Fluoline G) (BioMérieux, France) diluted 1/200. The threshold titre for positivity was set at 1:80.

### rK39-ICT serological test

The rK39-ICT test (Leti Laboratories, Spain) is a rapid, commercial, immunochromatographic test for the quantitative detection of *Leishmania* antibodies in serum. Serum (25 μl) was added to the test strips, along with the provided buffer solution, in 2 ml Eppendorf tubes. After 10 min at room temperature, the strips were examined for the two bands (control and specific) indicating a positive result.

### DNA extraction

DNA was extracted from 200 μl whole blood to which had been added 400 μl of NET10 (10 mM NaCl, 10 mM EDTA, 10 mM Tris HCl), 40 μl of SDS sample buffer (10%), and 2 μl of proteinase K. Samples were incubated with agitation overnight at 56°C. The DNA was isolated using the phenol-chloroform method, precipitating in ethanol [30]. The total DNA was resuspended in 100 μl of sterile distilled water and quantified using a UV-V ND-100 spectrophotometer (NanoDrop Technology, USA).

### *Leishmania* nested PCR

The extracted DNA was subjected to nested PCR (Ln-PCR) using primer pairs that amplify the *Leishmania* small ribosomal subunit (SSUrRNA) [31], employing a GenAmp PCR System 2700 thermocycler (Applied Biosystems, USA). The first round of reactions (30 cycles, annealing temperature 60°C) involved the use of primers R221 and R332. The amplicons were diluted 1/40 in distilled water, and 10 μl of this dilution used in the second round of reactions, which involved the use of primers R223 and R333 (30 cycles, annealing temperature 65°C). The amplicons were then visualised in 1.5% agarose gels in TAE buffer (Tris-acetate 0.04 mM, EDTA 1 μM, pH 8) using 0.02% GelRed staining (Biotium, USA) under a MiniBis-pro illuminator (DNR, Bio-imaging systems, Israel). Positive results require amplicons of 358 bp be detected.

### Statistical analysis

Normality was examined using the Shapiro-Wilk test. The Mann-Whitney U test was used to analyse differences between unpaired groups. Significance was set at P<0.05. The cut-offs for the ELISA and cytokine/chemokine release assays were determined by calculating the area under the receiver operating characteristic curve (AUC) and the 95% confidence intervals (CI). Spearman correlation coefficients were calculated between CD4+ T cells and SI, IFN-γ, IL-2 or MIG, and between SI and WBA-associated IFN-γ or IL-2. All calculations were undertaken using GraphPad Prism v.7 software (GraphPad Software, USA).

## Results

### The PBMC of HIV+ patients can mount a *Leishmania-*specific lymphoproliferative response

**Fig 1:**
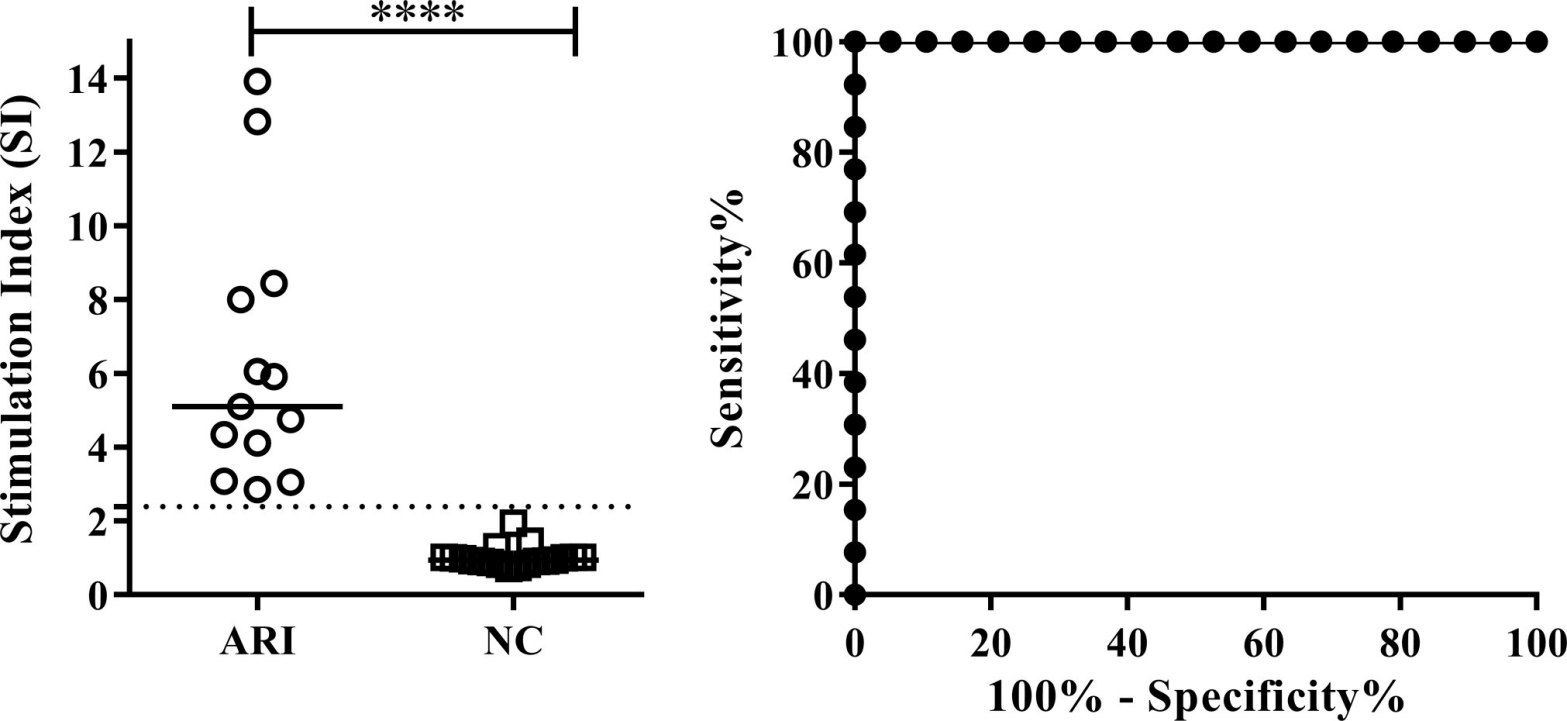
Lymphoproliferative response of HIV+ subjects living in the VL endemic area of Fuenlabrada following stimulation of their PBMC with SLA. A) Response to SLA by asymptomatic immune responders (ARI; n = 13), and non-responders (NC; n = 19). B) Area under the receiver operating characteristic curve. ****p<0.0001.

Among the present HIV+ subjects, none of whom showed any clinical manifestation of *Leishmania* infection, there was a group of 13 (15.85%) asymptomatic immune responders (ARI subjects) with a stimulation index (SI) of ≥2.39 in the SLA-CPA test (Fig 1A). The median SI of these 13 subjects was 5.11 compared to 1.02 for the non-responders (NC) (p<0.0001). The AUC for lymphoproliferation was 1.00 (95% CI: 1.00–1.00; p<0.0001); the sensitivity and specificity of the SLA-CPA test was therefore 100% (Fig 1B).

Following stimulation of the PBMC of all subjects with PHA-M, no significant difference was seen between the ARI and NC groups in terms of response; both were well capable of responding to PHA-M (S2A Table).

No *Leishmania* DNA was detected in any blood sample from any HIV+ patient using molecular techniques. In addition, the IFAT and ELISA serological tests were unable to detect any asymptomatic subject infected with *L*. *infantum* (results below the cut-offs). The serum rK39-ICT test was negative for all subjects.

### CD4+ T cell numbers were below the normal range in HIV+ subjects showing a cellular immune response to *Leishmania*, although these cells remained functional

No significant differences were seen between the ARI and NC subjects in terms of the size of their different lymphocyte populations (Table 1). Indeed, all populations were of normal size, except for that of the CD4+ T cells, which was below the normal lower limit (527/mm^3^).

**Table 1:** Median size (/mm^3^) of different lymphocyte populations in HIV+ subjects showing/not showing cell-mediated immunity to *Leishmania*.

	Median ± SEM					
	CD3+ T	CD8+ T	CD4+ T	B cells	NK cells	NK-T cells
ARI	1383.39 ± 155.70	916.9 ± 91.56	435.70 ± 70.53	202.42 ± 43.70	397.59 ± 78.86	85.10 ± 20.11
NC	1203.59 ± 226.10	877.35 ± 185.64	311.00 ± 56.33	131.4 ± 32.02	147.15 ± 33.87	67.92 ± 39.79

ARI: asymptomatic immune responders to *Leishmania*; NC: HIV+ patients with absent immune response to *Leishmania*; SEM: standard error of the mean.

**Fig 2:**
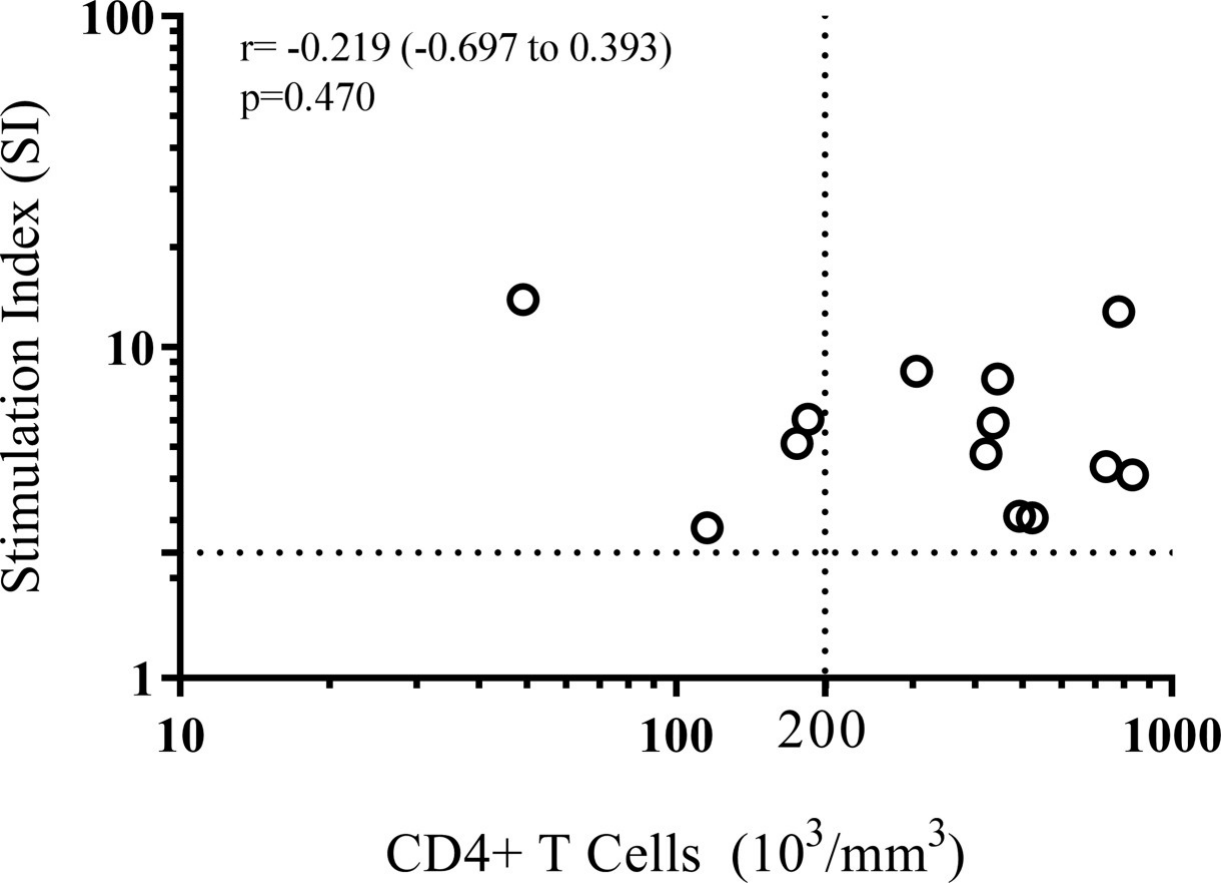
Specific lymphoproliferative response (stimulation index) *vs*. number of CD4+ T cells in HIV+ patients with cellular immune response to *Leishmania* (n = 13).

Four of the ARI subjects had a CD4+ T cell count of <200/mm^3^. However, all the ARI subjects were able to mount a cellular response to *L*. *infantum* (Fig 2, and S1 Fig).

### Asymptomatic immune responders to *Leishmania* showed increased cytokine/chemokine concentrations in cell culture supernatants

**Fig 3:**
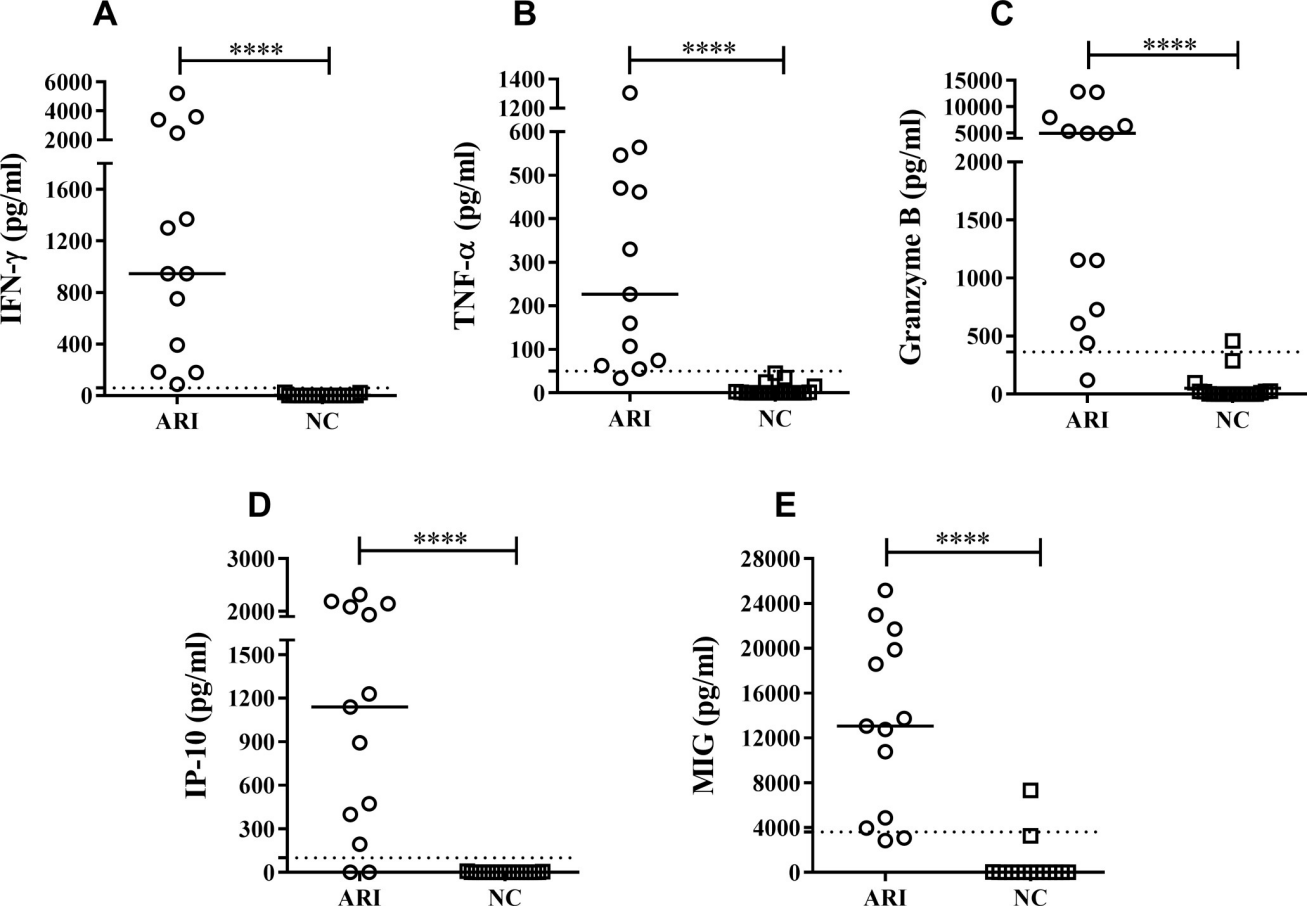
Production of cytokines and chemokines following the stimulation of PBMC from HIV+ patients with SLA for 6 days. ARI = asymptomatic immune responders to *Leishmania* (n = 13), NC = HIV+ patients with absent immune response to *Leishmania* (n = 19). Concentrations (pg/ml) of A) IFN-γ; B) TNF-α; C) granzyme B; D) IP-10; E) MIG. ****p<0.0001.

Following the SLA stimulation of the subjects' PBMC, the culture supernatants of the ARI subjects showed significantly greater median cytokine/chemokine concentrations than did those of the NC subjects: IFN-γ 946.2 *vs*. 0 pg/ml (p<0.0001), TNF-α 226.6 *vs*. 0 pg/ml(p<0.0001), granzyme B 668.7 *vs*. 0 pg/ml (p<0.0001) (Fig 3A–3C), IP-10 1139 *vs*. 0 pg/ml (p<0.0001) and MIG 13,062 *vs*. 9.13 pg/ml (p<0.0001) (Fig 3D and 3E). No IL-2 or IL-10 was detected in any supernatant for any subject.

Table 2 shows the high sensitivity and specificity of the increased cytokines and chemokines as markers of *L*. *infantum* cellular immune response in HIV+ persons. However, IFN-γ showed an AUC of 1.00 (95% CI; 1.00–1.00; p<0.0001): it therefore detected 100% of ARI subjects.

**Table 2:** Accuracy of detection of asymptomatic individuals via cytokine/chemokine analysis of supernatants from SLA-stimulated PBMC cultures.

Analytes	AUC	P value	Cut-off	Se (%)	95% CI	Sp (%)	95% CI
IFN-γ	1.000	<0.0001	> 56.29	100	75.29–100	100	86.77–100
TNF-α	0.9919	<0.0001	> 50.24	92.31	63.97–99.81	100	82.35–100
Granzyme B	0.9879	<0.0001	> 533.7	84.62	54.55–98.08	100	82.35–100
IP-10	0.9028	<0.0001	> 100.9	84.62	54.55–98.08	100	32.35–100
MIG	0.9692	<0.0001	> 3607	84.62	54.55–98.08	93.33	68.05–99.83

AUC: area under the curve; Se: sensitivity; Sp: specificity.

### Asymptomatic immune responders to *Leishmania* showed increased cytokine/chemokine concentrations in SLA-stimulated plasma from the whole blood assay

**Fig 4:**
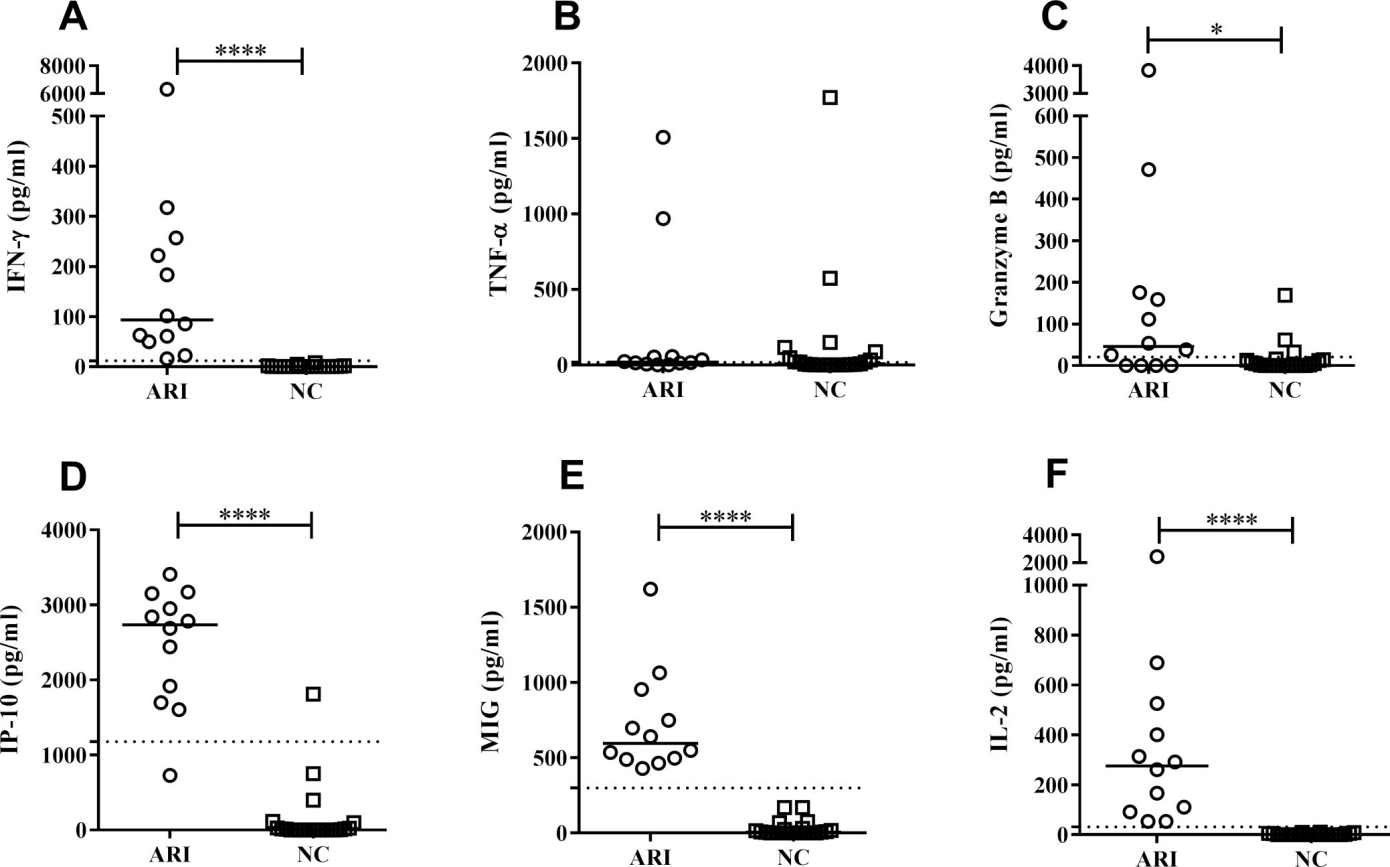
Cytokines and chemokines in SLA-stimulated plasma from HIV+ patients showing (ARI; n = 13) and not showing (NC; n = 19) cell-mediated immunity to *Leishmania*. Concentrations (pg/ml) of A) IFN-γ; B) TNF-α; C) granzyme B; D) IP-10; E) MIG; F) IL-2. *p<0.05 ****p<0.0001.

The SLA-stimulated plasma of the ARI subjects showed significantly greater median concentrations of certain cytokines and chemokines than did that of the NC subjects: IFN-γ 101.10 *vs*. 0 pg/ml (p<0.0001) (Fig 4A), granzyme B 53.64 *vs*. 1.68 pg/ml (p = 0.0396) (Fig 4C), IP-10 2785 *vs*. 2.27 pg/ml (p<0.0001) (Fig 4D), MIG 583.10 *vs*. 5.44 pg/ml (p<0.0001) (Fig 4E), and IL-2 291.90 *vs*. 0 pg/ml (p<0.0001) (Fig 4F). No significant differences were detected in the median production of TNF-α (22.61 *vs*. 6.93 pg/ml; p = 0.2610) (Fig 4B) or IL-10 (1.23 *vs*. 0.36 pg/ml; p = 0.7231).

Table 3 shows the sensitivity and specificity of the studied cytokines and chemokines as markers of *L*. *infantum* infection. The AUC of IFN-γ, MIG and IL-2 was 1.00 (95% CI 1.00–1.00; p<0.0001); these biomarkers therefore detect 100% of ARI subjects. The AUC for IP-10 was 0.985 (95% CI 61.52–99.79; p<0.0001), while those of TNF-α and granzyme B were more modest at 0.619 and 0.708 respectively.

**Table 3:** Accuracy of detection of asymptomatic individuals via cytokine/chemokine analysis of SLA-stimulated plasma from the whole blood assay.

Analytes	AUC	P value	Cut-off	Se (%)	95% CI	Sp (%)	95% CI
IFN-γ	1.000	<0.0001	> 11.67	100	76.84–100	100	84.56–100
TNF-α	0.619	0.2564	> 17.26	50	21.09–78.9	54.55	32.21–75.61
Granzyme B	0.708	0.0475	> 20.73	66.67	34.89–90.08	86.36	65.09–97.09
IP-10	0.985	<0.0001	> 1179	91.67	61.52–99.79	95.45	77.16–99.88
MIG	1.000	<0.0001	> 299.4	100	73.54–100	100	84.56–100
IL-2	1.000	<0.0001	> 31.66	100	73.54–100	100	84.56–100

AUC: area under the curve; Se: sensitivity; Sp: specificity.

The results show that all were capable of responding to PHA-M; no significant differences were seen between the ARI and NC groups (S2B Table).

### Strong responders in the proliferative assay were also strong responders in the whole blood assay

**Fig 5:**
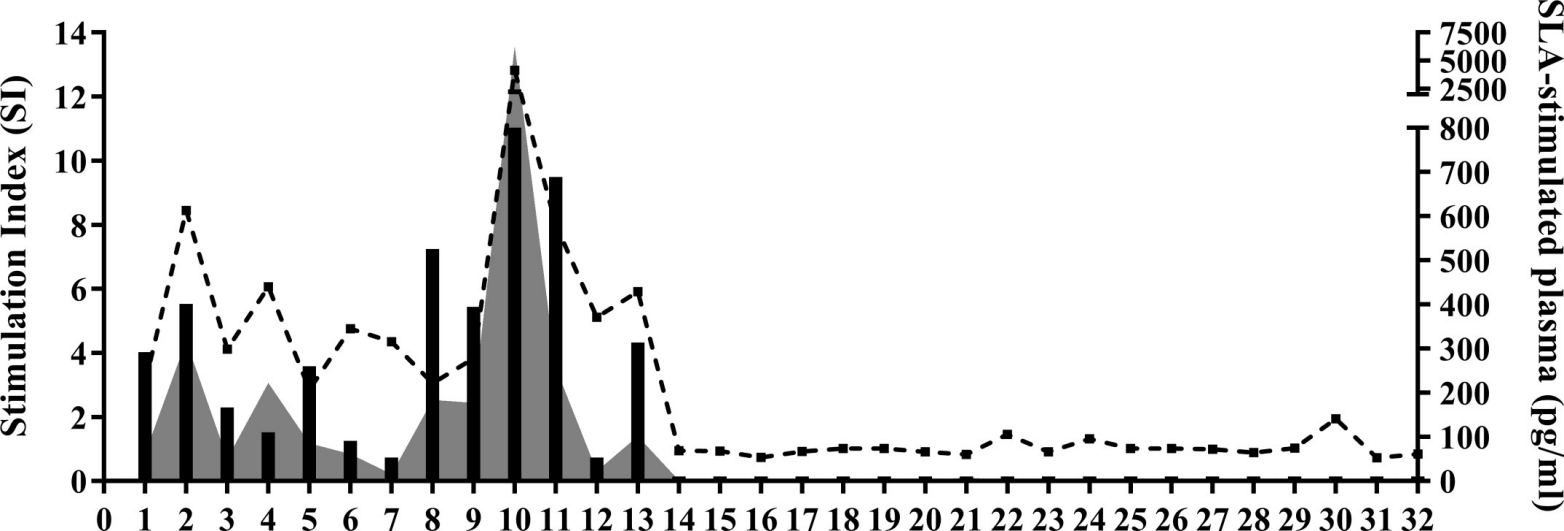
Stimulation index (dotted line) *vs*. IFN-γ (grey area) and IL-2 (black bars) production in SLA-stimulated plasma (WBA). The x-axis shows the individual subjects included in this study: 1–13 ARI and 14–32 NC.

A positive correlation was found between SI and IFN-γ, IL-2, IP-10 and MIG (as stimulated in the WBA) (Fig 5 and S3 Table).

## Discussion

Currently, no screening is undertaken to detect asymptomatic responders to *Leishmania* among persons who are HIV+, even though their risk of developing VL is relatively high. These carriers represent a risk to the success of *Leishmania* control strategies [32]. Field tools are therefore needed that can determine the real proportion of HIV+ patients that have been exposed to *Leishmania*. This is the first work using cytokine release assays to identify a sub-population of individuals who were exposed to *Leishmania* but in whom no clinical disease became manifest. This study also highlights a non-invasive, non-sensitizing simple assay of blood stimulation easily translatable to the field.

Cytokine release assays are useful for detecting asymptomatic individuals among immunocompetent subjects in VL-endemic areas; they can also detect the same among immunosuppressed subjects following solid organ transplantation [22, 24]. They are also useful for monitoring the success of treatment in HIV+ subjects [23]. From the present results, the concentration of IFN-γ in the supernatants of SLA-stimulated PBMC cultures appears as a major biomarker for both purposes. The present results show that the supernatants from the ARI subjects showed higher concentrations of IFN-γ than did those of the NC subjects, and that this cytokine is a 100% sensitive and specific biomarker of asymptomatic immune responders to *Leishmania* among persons who are HIV+.

The plasma IFN-γ, IL-2 and MIG concentrations following the SLA-stimulation of whole blood also identified 100% of these individuals. Unlike that reported for asymptomatic immunocompetent individuals, no differences were seen between the ARI and NC subjects with respect to TNF-α in the plasma of SLA-stimulated whole blood. This is probably due to the spontaneous production of TNF-α in HIV+ subjects [33, 34].

The present results show that the ARI subjects mounted a Th1-type cellular immune response to stimulation with SLA. However, the molecular and serological tests used were unable to detect *Leishmania* DNA or anti-*Leishmania* antibodies in peripheral blood respectively, as previously described for asymptomatic immunocompetent subjects from the same area [24, 25]. These results suggest that cellular immune tests should also be used when trying to identify such subjects. In line with our findings, IGRA positivity (WBA with stimulation by the antigens ESAT-6, CFP-10 and TB7.7, and quantification of IFN-γ) seems to be a better diagnostic tool for latent tuberculosis in HIV-infected patients than the Mantoux tuberculin skin test [35]. Further, the present HIV+ patients had CD4+ T cells numbers as low as 49/mm^3^. The capability to mount a specific cell immune response has also been described for HIV+ subjects co-infected with cytomegalus virus (with <350 CD4+ T /mm^3^) and *Mycobacterium tuberculosis* (with <100/mm^3^) [36, 37]. What does appear to be clear, is that HAART helps in maintaining them capable of mounting and/or maintaining a cellular immune response that might be involved in their asymptomatic status.

This work suffers from the limitation of a small sample size. Further studies should be performed with more subjects, and in different *Leishmania*-endemic areas to validate the use of the suggested biomarkers of asymptomatic immune responders to *Leishmania* in HIV+ patients. It would also be interesting to monitor the present ARI subjects to see whether they develop active VL, and how these biomarkers may change if they do. New studies are in progress to investigate these biomarkers during the asymptomatic period preceding the onset of active VL in HIV+ infected individuals from *L*. *donovani* endemic regions in Ethiopia.

The WHO guide for managing patients with leishmaniasis in Europe, published in 2017, recommends the use of the SLA-stimulated lymphoproliferation test, and WBA, plus subsequent cytokine/chemokine determinations for detecting cellular immune responses to *Leishmania* in immunocompetent patients [17]. The present work shows that these techniques are also valid for use with HIV+ patients living in a VL-endemic area.

In conclusion, the present results highlight the need to use cell immunity techniques to detect asymptomatic immune responders to *Leishmania* among HIV+ patients with no previous leishmaniasis or current symptomatology. Supernatants from SLA-stimulated PBMC cultures (CPA) can be used to look for IFN-γ, while SLA-stimulated plasma (WBA) can be used to look for IFN-γ, MIG and IL-2, all of which are biomarkers of the above condition. Combining these tests with molecular analyses could help to determine the true size of the *Leishmania* epidemic affecting the endemic area of Fuenlabrada and similar places. Some laboratory tests, including SLA-stimulated PBMC assay, may be difficult to perform under certain conditions. In contrast, the WBA holds much promise as a test at the point-of-care level.

## Supporting information

S1 Table Description of the HIV+ patients enrolled in this study. (DOCX)

S2 Table **Cytokines, chemokines, granzyme B production and stimulation index after PHA-M stimulation of PBMC (A) or whole blood (B) from HIV+ subjects showing an immune response to *Leishmania* (ARI) and without (NC)**.
(DOCX)

S3 Table Spearman correlation (r) between stimulation index (SI) and cytokines, chemokines and granzyme B from SLA-stimulated plasma (WBA). ****p<0.0001.
(DOCX)

S1 Fig Concentrations of cytokines/chemokines (pg/ml) vs. CD4+ T cell count in HIV+ subjects showing an immune response to *Leishmania* (n = 13). A) IFN-γ in supernatants of SLA-stimulated PBMC cultures (CPA); B) IFN-γ in SLA-stimulated plasma from the whole blood assay (WBA); C) IL-2 in SLA-stimulated plasma from the WBA; D) MIG in SLA-stimulated plasma from the WBA.
(TIF)
