# Supplementary material for: Asymptomatic immune responders to Leishmania among HIV positive patients
Source: PLoS Negl Trop Dis. 2019 Jun 3;13(6):e0007461. doi: 10.1371/journal.pntd.0007461 (PMC6564048; doi:10.1371/journal.pntd.0007461)
Supplement: S1 Table — (DOCX) [file pntd.0007461.s001.docx]

**Supplementary table 1.** Description of the HIV positive patients enrolled in this study.

| **Patients** | **Age (years)** | **Sex** | **Country of birth** | **HIV risk group** | **HIV diagnosis (year)** | **Start of HAART (year)** | **Start of actual HAART (year)** | **CD4 (mm^3^)** | **Viral load (copies/ ml)** | **Nadir CD4 (mm^3^)** | **Type of HAART** |
| --- | --- | --- | --- | --- | --- | --- | --- | --- | --- | --- | --- |
| 1 | 42 | M | Spain | IVDU | 1989 | 1995 | 2008 | 493 | <20 | 116 | AZT+TDF+LPVr |
| 2 | 44 | F | Africa | Heterosexual | 2009 | 2009 | 2009 | 305 | <20 | 66 | TDF/FTC/EFV |
| 3 | 29 | M | Spain | Homosexual | 2008 | 2008 | 2008 | 833 | <20 | 383 | TDF/FTC/EFV |
| 4 | 55 | M | Spain | IVDU | 2002 | 2002 | 2013 | 184 | 383 | 7 | DRVr+RAL |
| 5 | 50 | M | Spain | IVDU | 1989 | 1989 | 2012 | 115 | <20 | 69 | TDF/FTC+DRVr+ABC |
| 6 | 42 | M | Spain | Homosexual | 2008 | 2011 | 2011 | 422 | <20 | 275 | ABC/3TC/EFV |
| 7 | 44 | F | Spain | IVDU | 1991 | 2005 | 2013 | 737 | <20 | 270 | TDF/FTC+ETV |
| 8 | 56 | M | Spain | IVDU | 1991 | 1991 | 2013 | 522 | <20 | 198 | ETV + RAL |
| 9 | 48 | M | Spain | IVDU | 1992 | 1997 | 2011 | 49 | <20 | 4 | ABC/3TC + ATVr |
| 10 | 52 | F | Africa | Heterosexual | 2010 | 2010 | 2010 | 781 | <20 | 11 | TDF/FTC/EFV |
| 11 | 22 | M | Spain | Homosexual | 2012 | 2012 | 2015 | 445 | <20 | 303 | TDF/FTC/RPV |
| 12 | 53 | F | Africa | Homosexual | 2007 | 2008 | 2008 | 175 | <20 | 46 | TDF/FTC/EFV |
| 13 | 49 | M | Spain | IVDU | 2006 | 2006 | 2015 | 436 | <20 | 2 | TDF/FTC/ETGc |
| 14 | 55 | M | Spain | IVDU | 2010 | 2011 | 2011 | 552 | <20 | 474 | TDF/FTC+ATVr |
| 15 | 48 | M | Spain | IVDU | 1985 | 2003 | 2014 | 767 | <20 | 110 | ABC/3TC + RAL |
| 16 | 37 | M | Peru | Homosexual | 2009 | 2009 | 2009 | 279 | <20 | 117 | TDF/FTC/EFV |
| 17 | 38 | F | Africa | Heterosexual | 2002 | 2002 | 2011 | 460 | <20 | 265 | TDF/FTC+LPVr |
| 18 | 56 | M | Spain | Unknown | 2014 | 2014 | 2014 | 84 | 411 | 109 | TDF/FTC+DRVr |
| 19 | 57 | F | Spain | Heterosexual | 2010 | 2010 | 2010 | 311 | <20 | 73 | TDF/FTC/EFV |
| 20 | 39 | M | Spain | Homosexual | 2008 | 2012 | 2012 | 176 | <20 | 275 | TDF/FTC/EFV |
| 21 | 68 | M | Spain | Heterosexual | 2009 | 2009 | 2014 | 658 | 32 | 57 | DRVr |
| 22 | 50 | M | Africa | Heterosexual | 2007 | 2007 | 2007 | 770 | <20 | 34 | TDF/FTC/EFV |
| 23 | 49 | F | Spain | IVDU | 1988 | 2006 | 2013 | 146 | 42 | 23 | ABC/3TC + RAL |
| 24 | 52 | M | Spain | IVDU | 1985 | 2004 | 2012 | 446 | <20 | 8 | TDF/FTC+DRVr |
| 25 | 52 | M | Spain | IVDU | 1996 | 1997 | 2014 | 417 | 50 | 357 | DRVr+ETV+MRC |
| 26 | 47 | F | Spain | Heterosexual | 1997 | 2007 | 2010 | 252 | <20 | 6 | TDF/FTC/EFV |
| 27 | 35 | M | Spain | Homosexual | 2013 | 2013 | 2013 | 97 | 289 | 27 | TDF/FTC+DRVr |
| 28 | 31 | M | Ukraine | IVDU | 1999 | 2013 | 2013 | 96 | <20 | 83 | TDF/FTC/EFV |
| 29 | 54 | M | Spain | IVDU | 1987 | 1996 | 2013 | 18 | 621 | 22 | TDF/FTC+DRVr |
| 30 | 44 | M | Spain | Homosexual | 2005 | 2005 | 2005 | 678 | <20 | 257 | TDF/FTC/EFV |
| 31 | 22 | M | Spain | Homosexual | 2013 | 2013 | 2013 | 317 | 57 | 245 | TDF/FTC/EFV |
| 32 | 52 | F | Spain | Heterosexual | 2004 | 2004 | 2006 | 91 | <20 | 40 | TDF/FTC/EFV |
