# Supplementary material for: Asymptomatic immune responders to Leishmania among HIV positive patients
Source: PLoS Negl Trop Dis. 2019 Jun 3;13(6):e0007461. doi: 10.1371/journal.pntd.0007461 (PMC6564048; doi:10.1371/journal.pntd.0007461)
Supplement: S2 Table — Cytokines, chemokines, granzyme B production and stimulation index after PHA-M stimulation of PBMC (A) or whole blood (B) from HIV+ subjects showing an immune response to Leishmania (ARI) and without (NC). (DOCX) [file pntd.0007461.s002.docx]

**Supplementary table 2.**

A

| Median ± SEM | | | | | | |
| --- | --- | --- | --- | --- | --- | --- |
|  | **IFN-γ** | **TNF-α** | **Granzyme B** | **IP-10** | **MIG** | **SI PHA** |
| ARI | 12379.27 ± 2326.56 | 149.44 ± 181.60 | 898.86 ± 3149.45 | 2807.95 ± 709.05 | 599.61 ± 140.29 | 4.99 ± 1.28 |
| NC | 9074.54 ± 9050.42 | 320.22 ± 164.00 | 5266.73 ± 2122.35 | 2651.42 ± 1850.07 | 581.30 ± 225.70 | 5.20 ± 0.97 |

B

| Median ± SEM | | | | | | |
| --- | --- | --- | --- | --- | --- | --- |
|  | **IFN-γ** | **TNF-α** | **Granzyme B** | **IP-10** | **MIG** | **IL-2** |
| ARI | 824.20 ± 617.70 | 1273.00 ± 734.90 | 3945.00 ± 1321.00 | 787.30 ± 747.50 | 3350.06 ± 3298.34 | 465.20 ± 526.70 |
| NC | 495.20 ± 508.89 | 805.00 ± 716.40 | 3104.00 ± 723.00 | 1247.68 ± 466.02 | 1904.21 ± 1456.83 | 290.61 ± 294.50 |
