# Supplementary material for: Asymptomatic immune responders to Leishmania among HIV positive patients
Source: PLoS Negl Trop Dis. 2019 Jun 3;13(6):e0007461. doi: 10.1371/journal.pntd.0007461 (PMC6564048; doi:10.1371/journal.pntd.0007461)
Supplement: S3 Table — ****p<0.0001. (DOCX) [file pntd.0007461.s003.docx]

**Supporting Information**

**S3 Table.**

| **Correlation** | **r** | **CI 95%** | **P value** |
| --- | --- | --- | --- |
| **SI vs IFN-γ WBA** | **0,7101** | **0,4719 to 0,8516** | **< 0,0001** |
| SI vs TNF-α WBA | 0,02008 | -0,3406 to 0,3756 | 0,9131 |
| SI vs Granzyme B WBA | 0,2747 | -0,09258 to 0,5762 | 0,1281 |
| **SI vs IP-10 WBA** | **0,681** | **0,4270 to 0,8354** | **< 0,0001** |
| **SI vs MIG WBA** | **0,6857** | **0,4342 to 0,8381** | **< 0,0001** |
| **SI vs IL-2 WBA** | **0,7497** | **0,5353 to 0,8733** | **<0,0001** |

SI= stimulation index; WBA= whole blood assay stimulated with SLA; r= Spearman correlation; CI= confidence interval.
