# Supplementary figures and images for: Asymptomatic immune responders to Leishmania among HIV positive patients
Source: PLoS Negl Trop Dis. 2019 Jun 3;13(6):e0007461. doi: 10.1371/journal.pntd.0007461 (PMC6564048; doi:10.1371/journal.pntd.0007461)

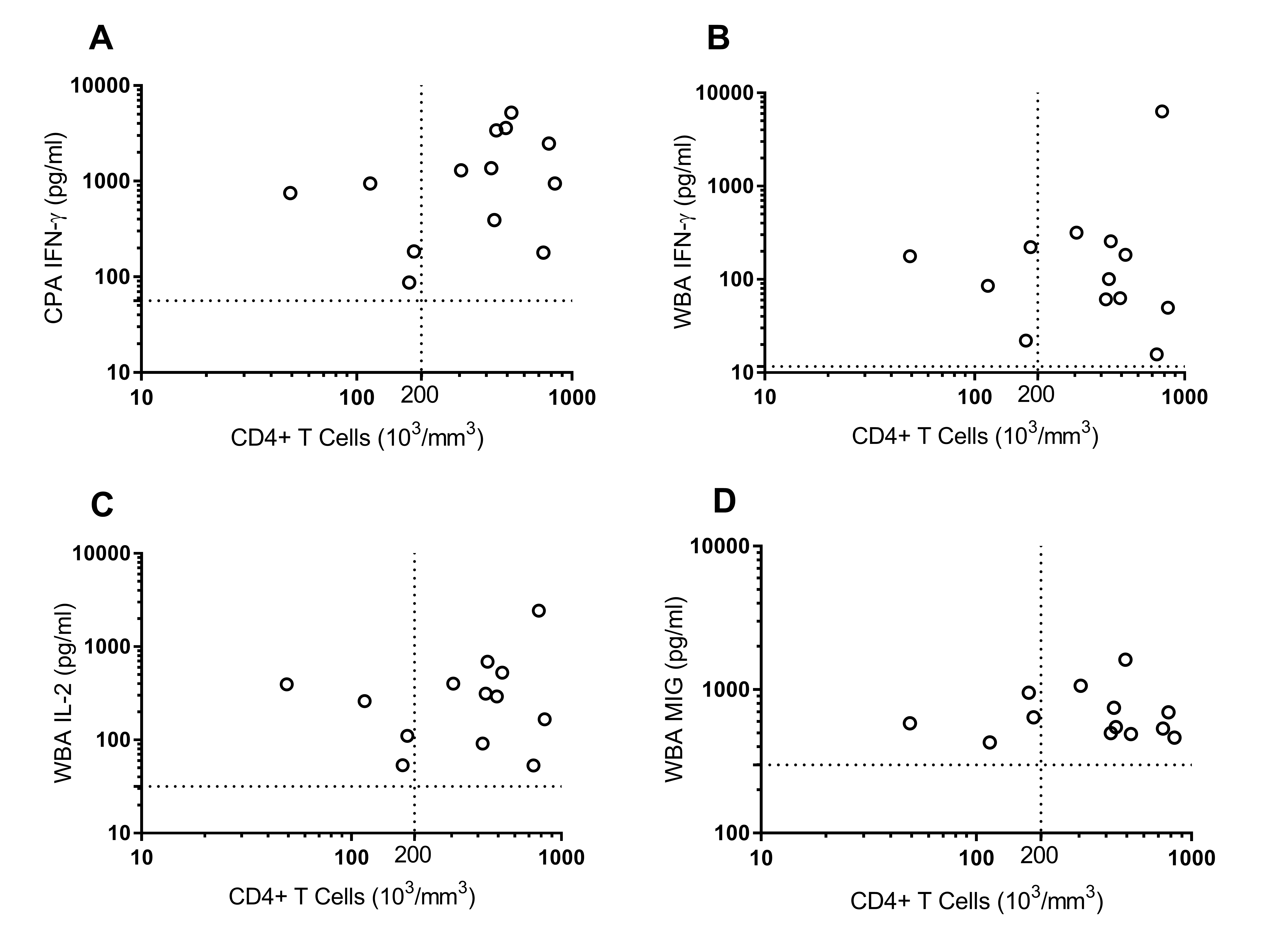

Supplement: S1 Fig — A) IFN-γ in supernatants of SLA-stimulated PBMC cultures (CPA); B) IFN-γ in SLA-stimulated plasma from the whole blood assay (WBA); C) IL-2 in SLA-stimulated plasma from the WBA; D) MIG in SLA-stimulated plasma from the WBA. (TIF) [file pntd.0007461.s004.tif]
